# Frequent Alteration of MLL3 Frameshift Mutations in Microsatellite Deficient Colorectal Cancer

**DOI:** 10.1371/journal.pone.0023320

**Published:** 2011-08-11

**Authors:** Yoshiyuki Watanabe, Ryan J. Castoro, Hyun Soo Kim, Brittany North, Ritsuko Oikawa, Tetsuya Hiraishi, Saira S. Ahmed, Woonbok Chung, Mee-Yon Cho, Minoru Toyota, Fumio Itoh, Marcos R. H. Estecio, Lanlan Shen, Jaroslav Jelinek, Jean-Pierre J. Issa

**Affiliations:** 1 Department of Leukemia, The University of Texas M. D. Anderson Cancer Center, Houston, Texas, United States of America; 2 Division of Gastroenterology and Hepatology, Department of Internal Medicine, St. Marianna University School of Medicine, Kawasaki, Kanagawa, Japan; 3 Department of Pathology, Yonsei University Wonju College of Medicine, Wonju, Korea; 4 Department of Biochemistry, Sapporo Medical University, Sapporo, Hokkaido, Japan; Bellvitge Biomedical Research Institute (IDIBELL), Spain

## Abstract

**Background:**

MLL3 is a histone 3- lysine 4 methyltransferase with tumor-suppressor properties that belongs to a family of chromatin regulator genes potentially altered in neoplasia. Mutations in MLL3 were found in a whole genome analysis of colorectal cancer but have not been confirmed by a separate study.

**Methods and Results:**

We analyzed mutations of coding region and promoter methylation in MLL3 using 126 cases of colorectal cancer. We found two isoforms of MLL3 and DNA sequencing revealed frameshift and other mutations affecting both isoforms of MLL3 in colorectal cancer cells and 19 of 134 (14%) primary colorectal samples analyzed. Moreover, frameshift mutations were more common in cases with microsatellite instability (31%) both in CRC cell lines and primary tumors. The largest isoform of MLL3 is transcribed from a CpG island-associated promoter that has highly homology with a pseudo-gene on chromosome 22 (psiTPTE22). Using an assay which measured both loci simultaneously we found prominent age related methylation in normal colon (from 21% in individuals less than 25 years old to 56% in individuals older than 70, R = 0.88, p<0.001) and frequent hypermethylation (83%) in both CRC cell lines and primary tumors. We next studied the two loci separately and found that age and cancer related methylation was solely a property of the pseudogene CpG island and that the MLL3 loci was unmethylated.

**Conclusions:**

We found that frameshift mutations of MLL3 in both CRC cells and primary tumor that were more common in cases with microsatellite instability. Moreover, we have shown CpG island-associated promoter of MLL3 gene has no DNA methylation in CRC cells but also primary tumor and normal colon, and this region has a highly homologous of pseudo gene (psiTPTE22) that was age relate DNA methylation.

## Introduction

In colorectal cancer (CRC), a systematic analysis of 13,023 well-annotated human protein-coding genes revealed mutations in 69 candidate genes [1]. Among these, the histone methyltransferase gene mixed-lineage leukemia 3 (MLL3) was mutated in 6 cases. MLL3 is a member of the TRX/MLL gene family and maps to chromosome 7q36.1. It encodes a predicted protein of 4911 amino acids containing two plant homeodomains (PHD), an ATPase alpha/beta signature, a high mobility group, a SET (Suppressor of variegation, Enhancer of zeste, Trithorax) and two FY (phenylalanine tyrosine) rich domains. PHD and SET domains proteins are chromatin regulators and several of them are altered in cancer [2]. Inactivation of MLL3 in mice results in epithelial tumor formation, suggesting that it functions as a tumor-suppressor gene [3]. Also, MLL3 has been reported to be frequently deleted in myeloid leukemias [4], [5]. Moreover, other reports indicate somatic mutations in the MLL3 gene in glioblastoma and pancreatic ductal adenocarcinoma [6]. However, subsequent reports have not yet confirmed MLL3 mutations in colon cancer [7]. Thus, the role of MLL3 in the pathogenesis of colorectal neoplasia remains incompletely defined.

In this paper, we investigated MLL3 alterations in colon cancer and found a two isoform of MLL3 of which the longer isoform has a previously unrecognized CpG island overlapping the promoter. Moreover, we found new genetic alterations in CRC cell lines and also primary tumors.

## Materials and Methods

### Ethics Statement

This study was approved by University of Texas M. D. Anderson Cancer Center and Yonsei University Wonju Christian Hospital Institutional Review Board, and written informed consent was obtained.

### Cell Lines and Specimens

Eight colorectal cancer cell lines (DLD1, SW48, RKO, HCT116, CaCo2, SW620, LoVo and SW480) were obtained from the American Type Culture Collection (Manassas, VA). All cell lines were maintained in appropriate media containing 10% fetal bovine serum in plastic culture plates. DNA was extracted using the standard phenol chloroform method, and total RNAs were extracted from the harvested cells using the Trizol (Invitrogen, Carlsbad, CA) [8]. We studied 72 samples of primary colorectal tumors obtained from Yonsei University Wonju Christian Hospital (Wonju, Korea) and 54 samples of primary colorectal tumors and 8 adjacent normal- appearing tissues from patients at M. D. Anderson Cancer Center (Houston, Texas). We also studied colonic biopsy specimens from 21 individuals with no family history of colorectal cancer and no colonic lesions at screening total colonoscopy.

### Mutation and DNA Methylation Analysis

DNA isolated from grossly microdissected cancers was analyzed to determine the somatic mutation of MLL3 using direct sequencing, and both methylation status of MLL3 and pseudo gene psiTPTE22 (pseudo-gene of transmembrane phosphatase with tensin homology on chromosome 22) using bisulfite pyrosequencing [9]. Direct sequencing analysis was conducted to identify mutations in all 59 MLL3 exons using both genomic DNA and cDNA of eight colorectal cancer cell lines, and confirm these sequences of mutation regions using genomic DNA of two different sets of primary CRCs (Table 1). Primer sequences were described in Table S1. All primers were synthesized by Invitrogen (San Diego, CA). PCR was performed in 22.5 µl Platinum PCR SuperMix High Fidelity (Invitrogen, San Diego, CA), 5 µM forward and 5 µM reverse primers and 10 ng genomic DNA. Reactions were carried out in 96 well MJ thermocyclers (MJ Research, Waltham, MA) using 30 cycles of PCR amplification protocol (denature 94c for 30 seconds; anneal 55c for 30 seconds; extend 68c for 60 seconds). PCR products were directly sequenced in the M. D. Anderson Core Sequencing Facility.

**Table 1:** Clinicopathologic features in Test Set and Validation Set.

Test Set			
Age	M (n = 49)	57.6±12.2	*p* = 0.62
	F (n = 23)	59.5±14.0	
Location	Restum	35	
	Sigmoid	37	
Histological Findings			
	Well Differentiated adenocarcinoma	6	
	Mod. Differentiated adenocarcinoma	44	
	Poor. Differentiated adenocarcinoma	14	
	Mucinus carcinoma	6	
	Unknown	2	
TNM Stage			
	T1	2	
	T2	4	
	T3	58	
	T4	8	
MLL3 Frameshift mutations (c.8382delA)			
(n = 72)	MSI	22.9% (8/35)	*p* = 0.04**
	MSS	5.4% (2/37)	
	N/D	0	

Validation Set			
	M (n = 36)	65.6±14.2	*p* = 0.81*
	F (n = 17)	65.4±15.3	
	Distal colon	18	
	Proximal colon	25	
	unknown	11	
UICC Stage			
	Stage 0	1	
	Stage 1	3	
	Stage 2	19	
	Stage 3	9	
	Stage 4	7	
	unknown	15	
MLL3 Frameshift mutations (c.8382delA)			
(n = 54)	MSI	28.6% (4/14)	*p* = 0.003**
	MSS	0% (0/40)	
	N/D	0	

*Student-t test.

**Fisher exact test.

UICC Stage: International Union Against Cancer.

N/D: non detectable.

Separate DNA methylation analysis between MLL3 and psiTPTE22 gene CpG islands were confirmed by bisulfate direct-sequencing after TOPO-TA cloning both colorectal cancer cell lines and primary CRCs. Information on mismatch repair (MMR) deficiency in cell lines was collected from published papers [10], [11] and the database of the Sanger Institute Cancer Genome Project (http://www.sanger.ac.uk/genetics/CGP/MSI/msi_page.shtml). In the primary colorectal cancer samples, we determined mismatch repair deficiency by microsatellite instability (MSI) analysis, as previously reported [12]. All primer sequences and PCR conditions are described in Table S1.

### Reverse Transcription-Polymerase Chain Reaction

**Figure 1:**
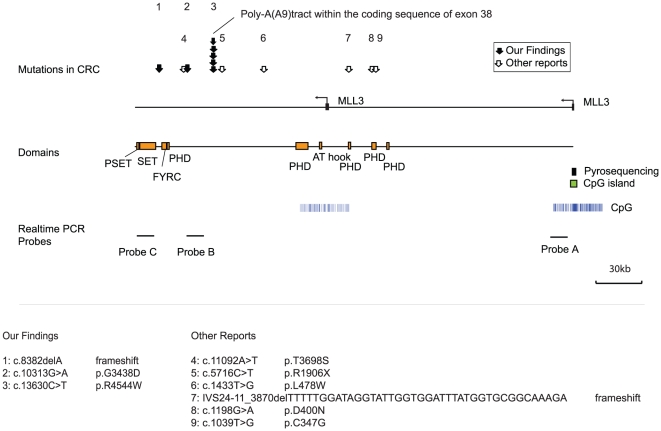
Genomic structure of the human MLL3 gene. MLL3 is transcribed from two separate promoters (arrows), and the promoter for the larger transcript contains a CpG island while there is none in the truncated form. The locations of mutations found in this and previous reports are indicated by arrows. Each arrow corresponds to a single case with a mutation, except for the one region with multiple arrows, which corresponds to the polyA tract. MLL3 gene encodes a predicted protein of 4911 amino acids containing two plant homeodomains (PHD), an ATPase alpha/beta signature, a high mobility group, a SET (Suppressor of variegation, Enhancer of zeste, Trithorax) and two FY (phenylalanine tyrosine) rich domains. Each of the mutations in primary samples were shown under the genome structure schema in Figure 1.

First-strand cDNA was prepared by reverse transcription of 1 µg samples of total RNA using Superscript III reverse transcriptase (Invitrogen, Carlsbad, CA). Real-time quantitative reverse transcription-PCR was carried out using Taqman Gene Expression Assays [MLL3 exon boundary 1 -2, Hs01005501_m1 (Probe A); MLL3 exon boundary 38 -39, Hs01005520_m1 (Probe B); MLL3 exon boundary 58 -59, Hs01005539_m1 (Probe C) and glyceraldehyde-3-phosphate dehydrogenase, Hs_00266705_gl (Applied Biosystems)] in colorectal cancer cell lines. (Figure 1). Human colon cDNA (BioChain, Hayward, CA) were used as normal controls; they were prepared from normal colon mucosae pooled from healthy subjects.

### Western Blotting

Rabbit polyclonal anti-MLL3 antibody (SAB1300328 Sigma-Aldrich, St. Louis, MO)) was used for immunoblotting. Whole cell lysates were prepared by scraping cell monolayers into assay buffer without SDS [containing 150 mmol/l NaCl, 50 mmol/l Tris–HCl (pH 7.2), 1% deoxycholic acid, 1% Triton X-100, 0.25 mmol/l EDTA (pH 8.0), protease and phosphatase inhibitors, 5 µg/ml leupeptin, 5 µg/ml aprotinin, 1 µg/ml pepstatin A, 1 mmol/l phenylmethylsulfonyl fluoride, 5 mmol/l NaF, and 100 µmol/l sodium orthovanadate], and protein concentrations were determined (Lowry reagent, Bio-Rad, Hercules, CA). Equal amounts of protein were separated by SDS-PAGE and transferred to PVDF membranes (Invitrogen, Carlsbad, CA).

### Statistical Analysis

Methylation levels obtained by pyrosequencing (%) were analyzed as a continuous variable for comparison of MLL3 gene methylation with clinicopathologic features; mean and 95% confidential intervals (CIs) were calculated. Two-sided *P*<0.05 was considered significant. All statistical analyses were performed using PRISM 4 software (GraphPad Prism, Inc., San Diego, CA).

## Results

In this study, we found frequent inactivation of MLL3 by a frameshift mutations in microsatellite deficient CRCs and no DNA methylation at the MLL3 loci in any colon samples.

**Figure 2:**
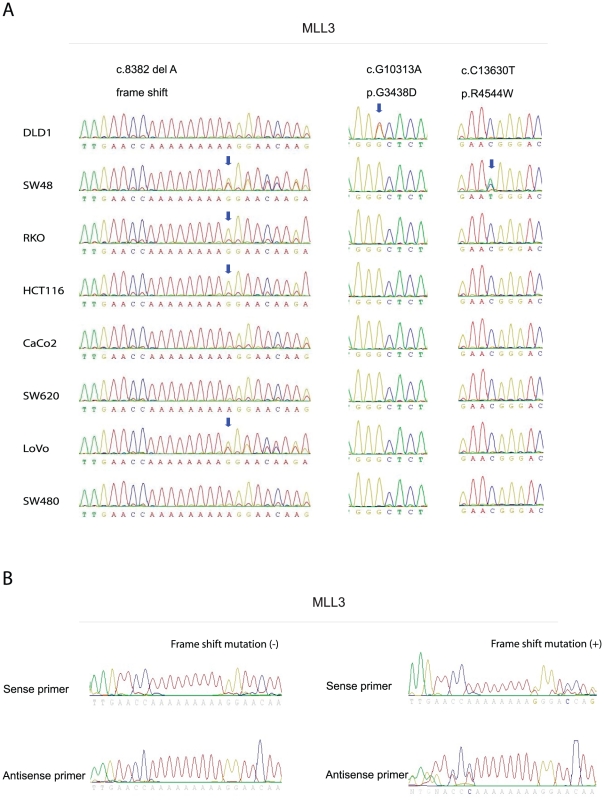
MLL3 mutation analysis in 8 colorectal cancer cell lines and 72 samples of primary colorectal tumors. (A) MLL3 has a poly-A(A)_9_ tract within the coding sequence of exon 38. Homozygous frameshift mutations were found in RKO and HCT116, while heterozygous mutations were found in the microsatellite unstable cell lines SW48 and LoVo. Separate somatic mutations were found in SW48 and DLD1 c.10313G>A (p.G3438D), c.13630C>T (p.R4544W). (B) Heterozygous mutations were found in the same poly-A(A)_9_ tract within the coding sequence of exon 38.

For mutation analysis, we screened 8 CRC cell lines using PCR and sequencing of all 59 coding exons. We found mutations in 5 out of the 8 cell lines (63.0%). MLL3 has a poly-A (A)_9_ tract within the coding sequence of exon 38 that is included in the processed transcript; homozygous frameshift mutations were found in RKO and HCT116, while heterozygous mutations were found in the microsatellite unstable cell lines SW48 and LoVo. These are all microsatellite unstable cell lines. Additionally, we found that SW48 and DLD1 have separate somatic mutations in other coding regions of MLL3 (Figure 1, 2A). These results of mutation analysis could confirm using cDNA (data not shown). Next, we analyzed the 3 somatic mutation regions (c.8382delA (frameshift), c.10313G>A (p.G3438D), c.13630C>T (p.R4544W)) in an initial set of 72 primary CRCs and found frameshift mutations within the (A)_9_ tract in 10 samples (MSI-H, 22.9% (8/35); MSS, 5.4% (2/37)) (Table 1). We then analyzed 9 somatic mutation regions including the 3 sites we found and 6 sites found by Sjoblom et al. [1] in a separate set of 54 primary CRCs and 21 healthy patient samples (Table 1). We found frequent mutations within the (A)_9_ tract in microsatellite unstable CRCs (28.6%, 4/14, see examples in Figure 2B) but no other mutations in these samples. Thus, overall, we found mutations in 19/134 cases analyzed (14%). These mutations are detectable in a broad range of the coding sequence, with clustering in the poly(A) tract, confirmed by our analysis of microsatellite unstable tumors.

**Figure 3:**
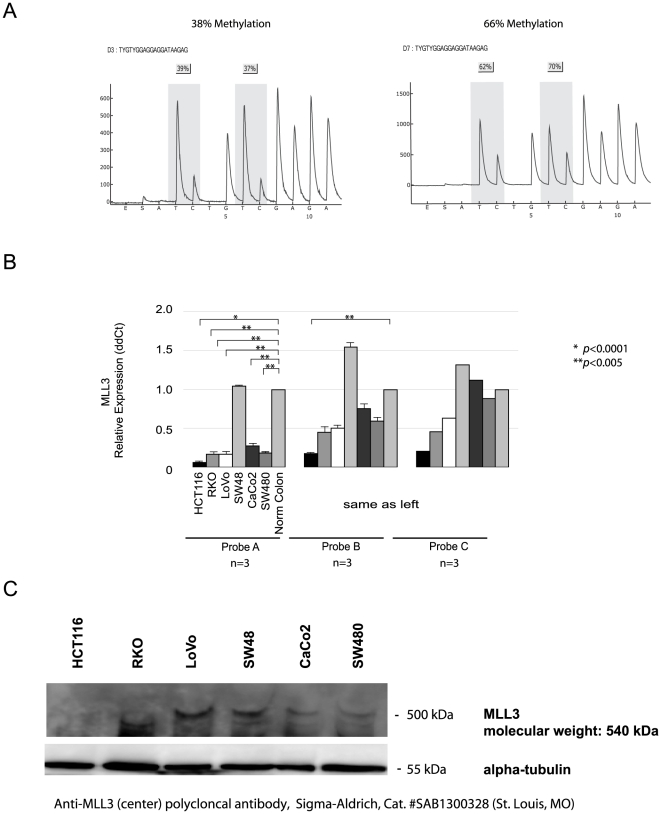
DNA methylation analysis using quantitative bisulfite pyrosequencing in primary CRCs and MLL3 relative expression, protein analysis in different CRC cell lines. (A) Example of pyrogram results using CpG island (primer region for pyrosequencing was shown in Figure 1), with polymorphic position C/T highlighted. Sequence reads TC/TGTC/TGGAGGAGGATAAGAG, Pyrogram in left side shows.normal colon and primary CRC (Right side). (B) Result of relative expression in normal colon and colon cancer cell lines analyzed by qPCR for the full length transcript (Probe A: Hs01005501_m1) and a mixture of the full-length and truncated transcripts (Probe B and C: Hs01005520_m1, Hs01005539_m1). Relative expression in probe A was down-regulated in 5 out of 6 cell lines examined. And the two other probes (Probe B and C) demonstrate minimal down-regulation (or even up-regulation) in these cell lines.

To study DNA methylation, we first noted that MLL3 is transcribed from two separate promoters, one of which results in a truncated version of the protein (Figure 1). The promoter for the larger transcript contains a previously unrecognized CpG island, but one is not present in the truncated form. We analyzed the methylation of this CpG island using quantitative bisulfite- pyrosequencing (examples in Figure 3A). However, we found that the CpG island of MLL3 area is highly homologous (∼92%) to a CpG island which overlaps the promoter of a pseudo- gene on chromosome 22 (psiTPTE22, pseudo-gene of transmembrane phosphatase with tensin homology on chromosome 22: NR_001591). Indeed the bisulfite pyrosequencing assay was able to amplify this region as well indicating the possibility of false positives. We found dense methylation in all 8 cell lines examined by pyrosequencing (HCT116, 74%: RKO, 66%: LoVo, 77%: SW48, 50%: CaCo2, 79%: SW480, 65%: DLD1, 72%: SW620, 74%), and a high degree of methylation in primary CRCs (45 out of 54 examined or 83.3%, Figure 3A). Methylation of this CpG island in cancer was not associated with common clinicopathologic features including age, gender, location and clinical stage. A measurable degree of methylation was present in the adjacent normal appearing mucosa of most patients analyzed, suggesting that this locus could be a target of age-related methylation [13]. Indeed, in healthy appearing normal colon mucosa samples, we found a strong age-related methylation of this CpG island (*r* = 0.88, *p* = 0.0001).

**Figure 4:**
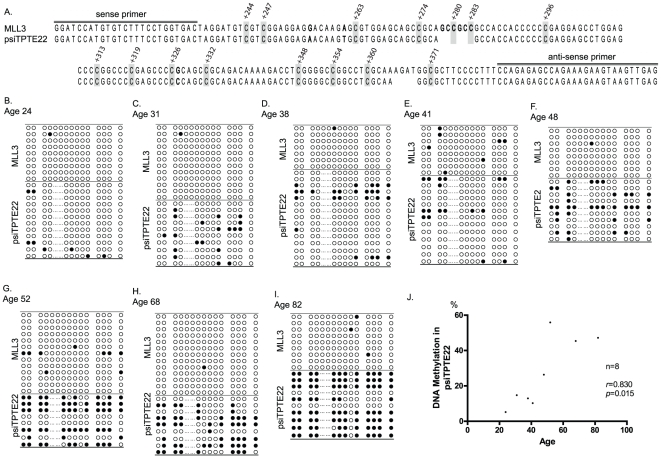
Methylation analysis by bisulfite direct-sequencing. (A–I) Bisulfite direct-sequencing was performed using primers that cover the promoter region of both MLL3 (larger form) and psiTPTE22 using different age of normal colon epitheliums (Age: 24, 31, 38 41, 48, 52, 68 and 82 years old). (J) Dots plotting shows association between DNA methylation in psiTPTE22 and each sample age. Results show that psiTPTE22 methylation was correlated with aging but not MLL3 (*r* = 0.830, *p* = 0.015).

We next sought to better understand the DNA methylation of the two homologous loci by performing bisulfite direct sequencing assays which could discriminate against the two loci. Because of the psiTPTE22 gene is 10 base pairs smaller than MLL3 gene in that region (Figure 4A). Interestingly, methylation of the MLL3 gene ranged from 0–5% in normal mucosa and CRC cell lines except for RKO (14.7%). However the, psiTPTE22 gene was highly methylated in colon samples, both in CRC cell lines and primary tumors. Additionally, methylation of the psiTPTE22 loci was associated with age related methylation in normal colon mucosa (Figure 4J).

To examine the expression profile of MLL3, we used qPCR (quantitative polymerase chain reaction) and three different Taqman probes to cover the full length transcript (NM 170606) and the truncated transcript (NM 021230) (Figure 1). As shown in Figure 3B, the full length transcript (Probe A) is substantially down-regulated in 5 out of 6 cell lines examined, suggesting a genetic etiology for this silencing (Figure 3B). By contrast, the two other probes (Probe B and C), which detect both truncated and full-length transcripts, demonstrate minimal down-regulation (or even up-regulation) in these cell lines. Collectively, the data show that somatic mutations particularly frameshift mutations in cancer silences the full length transcript while leaving the truncated transcript intact.

We next analyzed two different size of protein of MLL3 in cells (both wild type/frame shift mutation). Protein analysis was conducted by western blot to determine whether these cells can produce the appropriate MLL3 protein (Figure 3C). MLL3 has a truncated form in the 3′ end. Thus, we analyzed the MLL3 protein level using antibodies (this antibody was designed from center boundary of MLL3 (Sigma-Aldrich, Cat. #SAB1300328 (St. Louis, MO)). It will detect only long isoform of MLL3 protein product.

We found the appropriate band in CaCo2 and SW480, wild type of MLL3, and LoVo and SW48, heterozygous for the frameshift mutation, by MLL3 antibody. In contrast, there was no detectable band in RKO and HCT116, both homozygous for the frameshift mutation. These results correlated with the gene expression levels and protein analysis of MLL3 (Figure 3B, C).

## Discussion

In this study, we found frequent inactivation of MLL3 by frameshift mutations which had not been previously reported.

We have shown that the (A)_9_ tract in MLL3 is mutated in mismatch repair deficient tumors. A previous study [1] excluded mismatch repair deficient tumors and still found mutations in 2.2% of cases (6/37). In primary tumors, however, we screened for mutations in the previously reported affected regions and found only polyA tract mutations. We have thus underestimated the precise mutation rate of the gene given that we did not sequence all 59 exons in all tumors. Nevertheless, it is clear that MLL3 mutations resemble those of other important tumor-suppressor gene in CRC – TGFBRII. For both genes, most mutations seen in CRC are polyA tract mutations in mismatch repair deficient cases [14], but a few of the mutations are also found outside the polyA tract, including in cases without mismatch repair deficiency. Improvements in sequencing technologies and costs should allow the precise estimation of MLL3 mutations in primary CRCs in the near future.

Combination of epigenetic and genetic silencing characterizes several tumor-suppressor and cancer-predisposing genes such as P16, MLH1, VHL and others. MLL3 deceptively showed DNA hypermethylation in CRC cells but also in primary tumors by quantitative bisulfite pyrosequencing analysis. For this assay analyzed DNA methylation of CpG sites +224 bp from the TSS which indicated methylation in CRC cell lines, primary tumors and normal colon. However, we found that around the TSS of MLL3 there is a highly homologous (∼92.0%) to a pseudo gene on chromosome 22 which is named psiTPTE22 (pseudo-gene of transmembrane phosphatase with tensin homology on chromosome 22). TPTE (transmembrane phosphatase with tensin homology) is located on human chromosome 21 and has many homologous copies/pseudogenes on chromosome 13, 15, 21, 22 and Y [15].

We next analyzed these CpG site using bisulfate direct sequencing after TOPO-TA cloning in CRC cell lines (Figure S1A–I)) and primary normal colon samples (Figure 4A–J)). Bisulfite direct sequencing assay was able to distinguish the psiTPTE22 from MLL3 after sequencing because the promoter region of psiTPTE gene is 10 bp smaller than MLL3 gene (Figure 4A). We found that MLL3 showed only 0–5% methylation except for RKO which was methylated at 13.0%. On the other hand, psiTPTE22 was methylated between 65–90% in all CRC cell lines (Figure 4B–I). Addtionally, psiTPTE22 had methylation levels in normal colon tissues similar to what we previously observed for several genes [16].

Pseudogenes are defunct relatives of known genes that have lost their protein-coding ability or are otherwise no longer expressed in the cell [17]. Although some do not have introns or promoters, most have some gene-like features (such as promoters, CpG islands, and splice sites), they are nonetheless considered nonfunctional, due to their lack of protein-coding ability resulting from various genetic disablements (stop codons, frameshifts, or a lack of transcription) or their inability to encode RNA. Pseudogenes are characterized by a combination of homology to a known gene and nonfunctionality. That is, although every pseudogene has a DNA sequence that is similar to some functional gene, they are nonetheless unable to produce functional final products [18].

Interestingly, Liang et al described that psiTPTE22-HERV is silenced by DNA methylation in not only GI cancers but also renal, liver and lung cancer [19]. And HERV-related sequences in psiTPTE22-HERV are mostly spliced out as introns from the transcripts, and the amino acid sequence of the 15 kDa protein is not a homologue to any retroviral proteins. These make the HERV-related psiTPTE22-HERV gene an ordinary somatic gene.

In summary, we report that MLL3 is inactivated in CRC by genetic alteration. In particular, we found that microsatellite unstable CRC cell liness have frequent frameshift mutations within an (A)_9_ tract coding region of MLL3 causing a loss of protein function, and a previous study reported on mutations outside this tract in microsatellite stable cancers. Moreover, the MLL3 promoter CpG island is highly homologous to a CpG island in the promoter region of a pseudogene psiTPTE22. psiTPTE22 was densely methylation in both primary CRCs and correlated with aging in normal epithelium but not MLL3 (Figure 4A–J). MLL3 loss of function may be a key feature of early CRC tumorigenesis.

## Supporting Information

Figure S1 **Bisulfite direct-sequencing analysis of both MLL3 and psiTPTE22 methylation status in 6 CRC cell lines.** (A–I) We found 0–5% methylation of MLL3 in all cell lines. By contrast, the pseudo-gene (psiTPTE22) was methylated between 65–90% in all cell lines.
(EPS)

Table S1 **Primers sequences used for bisulfite-pyrosequencing and direct-sequencing analysis.**
(DOC)
