# Supplementary material for: Frequent Alteration of MLL3 Frameshift Mutations in Microsatellite Deficient Colorectal Cancer
Source: PLoS One. 2011 Aug 11;6(8):e23320. doi: 10.1371/journal.pone.0023320 (PMC3154922; doi:10.1371/journal.pone.0023320)
Supplement: Table S1 — Primers sequences used for bisulfite-pyrosequencing and direct-sequencing analysis. (DOC) [file pone.0023320.s002.doc]

| **Supplementary Table 1. Primers and PCR conditions** | | |  |  |  |  |  |  |
| --- | --- | --- | --- | --- | --- | --- | --- | --- |
| **Primers for Bisulfite-Pyrosequencing** | | |  |  |  |  |  |  |
|  |  | **Forward Primer** |  | **Reverse Primer** | **PCR condition (161bp)** | | | |
|  | MLL3Me-F | TAGGAGGAGGATYGGGATTTATGTGTT | MLL3Me-R | ACCRACCCCRAAATCTTTTATCTA | 62℃(3), 60℃(4), 58℃(5), 56℃(28) | | | |
|  |  | **Sequencing Primer** |  |  | **far from TTS** | | | |
|  | MLL3Me-Seq | TTTTTGGTGATTAGGATG |  |  | 224bp |  |  |  |
|  |  |  |  |  |  |  |  |  |
| **Primers for Direct Sequencing using gDNA** | | |  |  |  |  |  |  |
|  |  | **Forward Primer** |  | **Reverse Primer** | **PCR condition** | | | |
|  | MLL3-F1 | GAGGAGGAACCCGAGCAG | MLL3-R1 | AAAGTAAAGTGTGCGGAGCC | 55℃(30) | | | |
|  | MLL3-F2 | AAGGAAGTGCTGGATTTAAGGG | MLL3-R2 | AACAAGGCAGATGGGAAACTTAG | same as above | | | |
|  | MLL3-F3 | TGCGGTAGAGAATAAAGATGCAC | MLL3-R3 | TCATCCCAACTAAACACAGAGG |
|  | MLL3-F4 | GCTTAGCTTGTGCTTTAGACATAATTT | MLL3-R4 | GGAGAACAGGAGAAACTGTTCAA |
|  | MLL3-F5 | TGGCAGTACATTGCATTTAAACAG | MLL3-R5 | CAGTCCGGTGGTGGTACTG |
|  | MLL3-F6 | TCACAAATACAGGATGCCATTG | MLL3-R6 | CCACCATACCTGGCCTAGAA |
|  | MLL3-F7 | GCACACGAAGCGCTAAATTG | MLL3-R7 | CTGGCAACGATCCCTCAG |
|  | MLL3-F8 | TTCATGATGCAAATCCTTTCA | MLL3-R8 | GCTCTTTAGACGACCAGAGCA |
|  | MLL3-F9 | AACTGTGCAGTTCCCAGGAC | MLL3-R9 | AGTTTGGGTTTGAACAGCCAC |
|  | MLL3-F10 | GGGCAACAGAGTAAGAGTCCAT | MLL3-R10 | GAAGCTGTAAGATGGTAGAGCAAA |
|  | MLL3-F11 | TCAGCAATGCCTTCTCTTTACC | MLL3-R11 | CAGGATCCTCTCTTCCTGATTAAAG |
|  | MLL3-F12 | TGGTGATGATTTGCCCTTACTC | MLL3-R12 | CCTAAGTGACTGCATCTTGGC |
|  | MLL3-F13 | AATAAGTGGTACAATGTTTCATGCTT | MLL3-R13 | CCTTCTTTAGATCCATTGTCAATTCT |
|  | MLL3-F14 | GGAACCTGAAACAGTGGTATCC | MLL3-R14 | GTTTGGACCGAGGTCTACCAG |
|  | MLL3-F15 | TTCCTTAGTTGCTGGCTTTGA | MLL3-R15 | AAGTGTTTCTTGGAAGCTCAATC |
|  | MLL3-F16 | TCACAGTGACAGGCCTAAGGT | MLL3-R16 | GGAGTCAAAGAGGAAGGTAAGAAA |
|  | MLL3-F17 | TGGAATTGTTATTCTAGACCTTAGCC | MLL3-R17 | AATCTTCCTTCTGCTCCTTGG |
|  | MLL3-F18 | CCCATTATGTTCGTATGTAGCTTCC | MLL3-R18 | CAGGAAATCGCTTCTGTAAGTTTC |
|  | MLL3-F19 | CAAGGTAGGAGGAGCATGTGA | MLL3-R19 | CAATCAACATATTGGATTCTGACA |
|  | MLL3-F20 | TTCCTCCATGACTTGAGTATTTCC | MLL3-R20 | TGAAGCAGGTTTGACAAGTGG |
|  | MLL3-F21 | TGCAACCTTTCTGCCTGTAAT | MLL3-R21 | ATCACTCATTTGGAAATGGCA |
|  | MLL3-F22 | ATGGCTCAGCAACAACCCTAC | MLL3-R22 | AACATTTGGGACAAGGCATTC |
|  | MLL3-F23 | AAGTGATAAGAGTGGCTTTGCC | MLL3-R23 | AGTAACAATAAGAGGCAATAAGGCTC |
|  | MLL3-F24 | TGAAACTGTGTGTATGTTGGTAAGG | MLL3-R24 | TGACAAGTATCAAAGCACACAAA |
|  | MLL3-F25 | TGTTATTTCTGCTTCCTTGTCAAA | MLL3-R25 | ATGCATATGAGGCCAAACAAA |
|  | MLL3-F26 | CTCACTCAGTATCTGCTGTACTCTAGG | MLL3-R26 | GAAAGCTGAGGCAGATCAAAG |
|  | MLL3-F27 | TGAGCGACTCTCCAAATTGAT | MLL3-R27 | TTTCCAAGAATTGCTCCTAGAAT |
|  | MLL3-F28 | TTTGCCCTACATCTAGGCTTTC | MLL3-R28 | CCCTGATTGTGTATTGGCAAC |
|  | MLL3-F29 | TTAATTTATTCTAGGAGCAATTCTTGG | MLL3-R29 | CGTGGCTACTAAAGAATAGGGC |
|  | MLL3-F30 | TTCAAGGGCCAGCTTTACTTC | MLL3-R30 | AAGCCACAATAAACGCAACATAC |
|  | MLL3-F31 | TGTATGTTGCGTTTATTGTGGC | MLL3-R31 | GGAATCTTCATGTTGTGGGTC |
|  | MLL3-F32 | CGGCCTGAATAAGATTAACTGTC | MLL3-R32 | AAAGTGAAATGAATTACTCCTTCCC |
|  | MLL3-F33 | GGGAGGTGCCGTAAGTATTTC | MLL3-R33 | CATTTGTGCCTAGGGCAGTAT |
|  | MLL3-F34a | CTGGTAGCGCTGACATCAAAC | MLL3-R34a | AGGTGGAGCTTGTGGCTTTAC |
|  | MLL3-F34b | TCTTCTGGTGCAGTCTGGTTC | MLL3-R34b | CTGAAACTACAGGAGACCGGG |
|  | MLL3-F34c | TTGTAAAGCCACAAGCTCCAC | MLL3-R34c | TCAAGAGGTGCAGGTGTCAAC |
|  | MLL3-F34d | ACAACAGCAAATAGGCCATCC | MLL3-R34d | GTTGGGAGTATGGGTCCTGAG |
|  | MLL3-F34e | TCAAGGCGATTGTCTGTTGAC | MLL3-R34e | CAGGAAAGGATCCTGATTTGG |
|  | MLL3-F34f | GACCCATATAGTCAGCAGCCC | MLL3-R34f | GGGAGTGCATTGGAGAGTTTG |
|  | MLL3-F34g | CAAATCAGGATCCTTTCCTGC | MLL3-R34g | CTCCTGAAGTTGGCACAGGTC |
|  | MLL3-F35 | GCAACATAGCAAGATCCCATATC | MLL3-R35 | AGGCACAAGCCACCACAC |
|  | MLL3-F36a | TGCTTGGTTGTTCTTCCTTTG | MLL3-R36a | TATGCTTGGCCCAGTATGTTG |
|  | MLL3-F36b | GTACCATGCCGAGTCAAGAGC | MLL3-R36b | CTAGACCATCAGAAGGCTGGG |
|  | MLL3-F36c | CCCAGGGTCTACCTAATCAGC | MLL3-R36c | TAAGAGGTCATCCAGGTTGGG |
|  | MLL3-F36d | AGATAACCACCCAGCCTTCTG | MLL3-R36d | CTGGATCACAAGGATGCAAAG |
|  | MLL3-F36e | GATCCAGAACTTGACATGGGAG | MLL3-R36e | CTTGACACATGATTGGATGGG |
|  | MLL3-F36f | AACCAATCGAGAAACTGCTGG | MLL3-R36f | GAATCATGGCTTGCATCTGTC |
|  | MLL3-F36g | AAACCTGCAACTCAAACTGGG | MLL3-R36g | AAACAAACTCCTCAAGGAGGAATAG |
|  | MLL3-F37 | TAAACCCAGGATAAGGTTGCC | MLL3-R37 | CATTCCTAGACAAGGGCTTTCC |
|  | MLL3-F38 | TGCCCAGTACATGAAAGGTAGG | MLL3-R38 | GCAAATGGCATATGGTGAAATC |
|  | MLL3-F39 | TCAGAATTAGGTTGCCACCAC | MLL3-R39 | TTTAATAAGACAGGAGGGCACC |
|  | MLL3-F40 | TGTGGTCATGGTGAAACTACTTC | MLL3-R40 | TGTTATGGCTGCAGTTACTAAGAGG |
|  | MLL3-F41a | GCCCTCTGTGTAACCTCAACC | MLL3-R41a | TTGGATCCGTTGTCTCTCTTG |
|  | MLL3-F41b | AATGCCCAGTTTACCTGGATG | MLL3-R41b | AGGGATTGATGGTGAATCAGG |
|  | MLL3-F41c | TTCAAGAACGGGAACGTAAGG | MLL3-R41c | TAGCTACTGGAGGTGCTGCTG |
|  | MLL3-F41d | TAATTCACCCTCCACCCAAAC | MLL3-R41d | GACTGGTTCCACCGACTCTTG |
|  | MLL3-F41e | TACCAGCAGCACCTCCAGTAG | MLL3-R41e | ACAGGCTACAGCGTTTCCTTC |
|  | MLL3-F41f | GCAACTCCAAATCAACAGACG | MLL3-R41f | TTCAGCTGGGTTCTGCTTCTC |
|  | MLL3-F41g | TGGTAGTAAGGTAGAAGGAAACGC | MLL3-R41g | TGGGTTCCAGAGAGGTAGAAATG |
|  | MLL3-F42 | ATAGCTTGGCACTGCATGAAC | MLL3-R42 | CATGCCTCAAGTGATCCACC |
|  | MLL3-F43 | CTGTAGCCTTGCTTCTATGTGC | MLL3-R43 | TGGCACATCAACTGTCTTGG |
|  | MLL3-F44 | GCCTCTGCCAGTTGTGTATTG | MLL3-R44 | GCTATTCCTAAAGACATTATGCCAC |
|  | MLL3-F45 | ATGTCAGCAGGGTTGGTACAG | MLL3-R45 | TGATGTCATTCCTTCTTGATTCTG |
|  | MLL3-F46 | CCATCATTCCAATTCTTCCTAGC | MLL3-R46 | GAGGTCTGCAGTGAGCATCAG |
|  | MLL3-F47 | TCATGCAAAGAGGATTTATCCTAAG | MLL3-R47 | TACGCCAGCATGTTACCTGTC |
|  | MLL3-F48 | ATACTGCCATTGCTTTCCCAG | MLL3-R48 | CCAGGAACATGTGTCCATCAC |
|  | MLL3-F49 | ACACCAAAGAGCTACAACAGGC | MLL3-R49 | TCACTCCCATATGCCCAGAAC |
|  | MLL3-F50a | CTCAGGTCACCCATCCTCTTC | MLL3-R50a | TTGCTGGTCCATCTGTCAATC |
|  | MLL3-F50b | CTCCACTTTGGATGTGCACTG | MLL3-R50b | ATGTTGGTGCATCGAAATCTG |
|  | MLL3-F50c | CTTAAACCTGATCCTGTGCCC | MLL3-R50c | CAATTGTGTGGAAGATGAGGC |
|  | MLL3-F50d | TAGTGGATGCCACAGATTTCG | MLL3-R50d | CCTTGTTCCACAATCCTGATG |
|  | MLL3-F50e | GGGTAGCCTCATCTTCCACAC | MLL3-R50e | AGAACACAAACATTGGCATTTG |
|  | MLL3-F51 | TGCCCAACAGGATTTATTTGG | MLL3-R51 | TGCACAGGCTCAGTCACTTTC |
|  | MLL3-F52 | TGGCTTTGAAACTGAATTTGC | MLL3-R52 | ACAGCAGGGTTTGTAAGCAGC |
|  | MLL3-F53 | GTCTCTGCTAGGCAGCTTTCC | MLL3-R53 | TCCCTGCAGCTATGTGAAATC |
|  | MLL3-F54 | ATAAGACCCGAGGGTGAAACC | MLL3-R54 | CAGATTTCATTCTGCCTTCCAG |
|  | MLL3-F55 | CAGTAAGGATGATCAGGCTGC | MLL3-R55 | TGAATGTTTAGTCACCGCCAG |
|  | MLL3-F56 | TTTGTGCTTCTAACAATGGGC | MLL3-R56 | GAGATACTGCAGGGTGGGAAC |
|  | MLL3-F57 | TCCCATGGAACCTCAATAAATC | MLL3-R57 | ACGCTGCTTCTGTCAGCTTC |
|  | MLL3-F1x1 | TGGAATTGTTATTCTAGACCTTAGCC | MLL3-R1x1 | TGGATGGTAACACTGACCACA |
|  | MLL3-F2x2 | ATAGCTTGGCACTGCATGAAC | MLL3-R2x2 | CATGCCTCAAGTGATCCACC |
|  | MLL3-F3x3 | TGCTTCGGGTCTTAGAAAGTTG | MLL3-R3x3 | AAGGAAATAAGGATTTCCACTACCA |
|  | MLL3-F4x4 | TGGCTTTGAAACTGAATTTGC | MLL3-R4x4 | ACAGCAGGGTTTGTAAGCAGC |
|  |  |  |  |  |  |  |  |  |
| **Primers for Direct Sequencing using cDNA** | | |  |  |  |  |  |  |
|  |  | **Forward Primer** |  | **Reverse Primer** | **PCR condition** | | | |
|  | MLL3cDNA-F | AGGAACTAGACCTTCCAATTGATGA | MLL3cDNA-R | TGTTTATCAGAGAGAACCAGAGTTTTG | 55℃(30) | | | |
